# Barriers to accessing aeroallergen immunotherapy in paediatrics

**DOI:** 10.1007/s11845-022-03067-x

**Published:** 2022-07-08

**Authors:** James Trayer, Aideen Byrne, Basil Elnazir

**Affiliations:** 1 Children’s Health Ireland at Tallaght University Hospital, Tallaght, Dublin 24, Ireland; 2 Children’s Health Ireland at Crumlin, Crumlin, Dublin 12, Ireland; 3 Trinity College Dublin, Dublin, Ireland

**Keywords:** Allergic rhinitis, Immunotherapy, Pollen allergy, Prescribing

## Abstract

**Background:**

Allergen immunotherapy (AIT) is a safe, effective and disease-modifying treatment for allergic rhinitis. It is indicated for children with moderate to severe disease whose symptoms persist despite conventional therapy. There is a high prevalence of allergic rhinitis amongst Irish children; however, levels of AIT prescribing in Ireland are lower than neighbouring countries.

**Aims:**

The aims of this study are to describe current patterns of AIT prescribing and referrals amongst Irish paediatricians and to identify barriers to accessing AIT in Ireland.

**Methods:**

An electronic questionnaire was distributed to all paediatricians and paediatric trainees caring for children with allergic rhinitis.

**Results:**

A lack of knowledge of AIT clinical criteria was the most frequently reported barrier with 50.5% (50/99) of general paediatricians unaware of the indications for referral compared to 27.3% (3/11) of respiratory physicians and 0% (0/8) of allergists. Accessibility is the next most cited barrier with 31.4% (37/118) of respondents unsure where to refer and 19.5% (23/118) reporting a lack of local services. Cost was reported to be a barrier by 12.7% (15/118). Paediatricians with an allergy or respiratory subspecialisation reported seeing significantly higher numbers of children with allergic rhinitis and were more likely to prescribe or refer a child for AIT.

**Conclusions:**

This study demonstrated positive attitudes towards AIT amongst all grades and specialities of paediatricians in Ireland. The main barriers to more widespread use are difficulties with the identification of suitable candidates by general paediatricians and a lack of local AIT services and referral pathways.

## Introduction

Allergen immunotherapy (AIT) was first described in 1911 [1]. It involves the regular exposure of a patient to a known allergen with the aim of inducing tolerance by modifying the immune process. A course of AIT lasts at least 3 years and can be administered either by subcutaneous injection (SCIT) or daily sublingual tablets/liquid (SLIT) [2].

AIT has been shown to be effective with a reduction in allergic rhinitis (AR) symptoms and medication usage during treatment [3]. This immunomodulatory effect is sustained after completion of 3 to 4 years of treatment [4, 5].

AIT is generally well tolerated though severe reactions including anaphylaxis have been described. Numerous international guidelines exist, including those by EAACI, BSACI and AAAAI [2, 6, 7]. Poorly controlled asthma is the main risk factor for severe reactions and is included in all major guidelines [2, 6, 8].

Asthma and AR frequently co-exist. AIT has been shown to reduce usage of conventional medications for AR in addition to inhaled corticosteroid use in patients with co-existing asthma [9, 10]. There is evolving evidence for additional disease-modifying benefits of AIT in children with grass pollen allergy as a treatment course may reduce their likelihood of developing asthma later in life [11]. Systematic reviews investigating cost-effectiveness of AIT demonstrated economic benefits after 6 years and a favourable cost per QALY [3, 12].

Irish children have a high prevalence of AR. A study of Irish schoolchildren demonstrated a prevalence of 23.5% for asthma and 10.6% for AR in children aged 6–9 years [13]. In children aged 13–14, the prevalence of AR is as high as 31.5% with 0.9% reporting severe rhinoconjunctivitis in the past year [14]. This represents a significant health burden amongst Irish children and symptom control is essential to prevent detrimental effects on sleep and school performance [15–17]. A national review of AIT services in Ireland showed that although AIT is available in centres throughout the country, there is low uptake with only 0.01% of potential patients receiving AIT [18]. In contrast, a German study described AIT use in 6–7% of patients with grass pollen allergy and up to 16% of those with house dust mite allergy [19]. Spanish studies of patients attending allergy services reported AIT use in 30 to 41% of those with grass and house dust mite allergy [20, 21]. A survey of Irish allergists performed as part of a wider European study identified accessibility and cost as the main barriers to accessing AIT in Ireland [22].

According to the Primary Care Reimbursement Scheme annual report 2019, a total of 3201 prescriptions representing a total of 266 patients were prescribed AIT in 2019. This number includes both children and adults entitled to free medications under national means-tested schemes and excludes patients treated in the private sector. Despite the limitations of this data, it suggests a very low rate of AIT prescribing in Ireland. Applying this number to the ISAAC study results and most recent Irish census data from 2016 suggests that even if all of the reported prescriptions were for children it still only represents 0.003% of children with AR and 0.01% of those with severe disease [14].

AIT is safe and effective and is the only disease-modifying treatment for AR. It should be considered where symptoms are uncontrolled despite conventional therapies. Rates of AIT prescribing in Ireland are significantly lower than neighbouring countries and this study aims to identify barriers to accessing AIT.

## Methods

A questionnaire was designed based on similar studies and international guidelines [2, 23]. The questionnaire contained demographic questions followed by sections assessing participants’ knowledge of AIT and attitudes regarding safety, cost-effectiveness and barriers towards AIT prescribing. Ethical approval was granted by the Joint Research and Ethics Committee of Tallaght University Hospital. The questionnaire was distributed electronically via the Royal College of Physicians in Ireland with a follow-up email via the hospital administrator in each paediatric centre in Ireland. The questionnaire was distributed over a 3-month period between December 2020 and March 2021. A participant information leaflet accompanied the questionnaire and a decision to participate was understood to imply consent. The target population was all paediatricians who work in a specialty where they manage children with AR. The total population was estimated at 285 physicians and a target of 100 responses was chosen. Data was analysed with SPSS 27 (IBM). Descriptive data is described in percent. Chi-square test for independence was used for comparisons between groups. Fisher’s exact test was used when the frequency of any cell was less than 5. A *p* < 0.05 was considered statistically significant. General paediatricians with a special interest in allergy or respiratory were included with dedicated allergists and respiratory physicians for analysis.

## Results

A total of 120 responses were received. One response was excluded as it had been completed by a subspecialist with no contact with patients with AR. Another was excluded as it was returned after the predefined data collection period. The remaining 118 responses represent 41.4% of the estimated population of interest and exceed the target of 100 responses. Respondent demographic data is described in Table 1.

**Table 1:** Demographic details of respondents

**Gender**			**Number (%)**
	Male		44 (37.3)
	Female		74 (62.7)
**Grade**			
	SHO		25 (21.2)
	Registrar		52 (44.1)
	Consultant		41 (34.7)
**Years qualified**			
	0–5 years		31 (26.3)
	6–10 years		36 (30.5)
	> 10 years		51 (43.2)
**Speciality**			
	**General Paediatrics**		99 (83.9)
	SHO	25 (25.3)	
	Registrar	49 (49.5)	
	Consultant	25 (25.3)	
	**Respiratory**^a^		11 (9.3)
	SHO	0 (0)	
	Registrar	1 (9.1)	
	Consultant	10 (90.9)	
	**Allergy**^b^		8 (6.8)
	SHO	0 (0)	
	Registrar	2 (25)	
	Consultant	6 (75)	

^a^Including general paediatrics with a special interest in respiratory

^b^Including general paediatrics with a special interest in allergy

### Knowledge of AIT amongst Irish paediatricians

The majority of respondents (93.2%, 110/118) were aware of AIT as a treatment option for AR in children. Despite this, only 60% (15/25) of general paediatric consultants were aware of local services compared to 90% (9/10) of respiratory consultants and all allergists (6/6).

Only 33.1% (39/118) of respondents were aware of AR guidelines. This varied significantly between specialities with all allergists, 54.4% (6/11) of respiratory physicians and just 25.3% (25/99) of general paediatricians aware of guidelines (*p* < 0.001). EAACI was the most well-known (59%) followed by BSACI (43.6%), ARIA (35.9%) and AAAAI (15.4%).

### Patterns of AIT referral and prescribing

Allergists and respiratory physicians reported seeing more children with asthma and AR than general paediatricians. 9.1% (9/99) of general paediatricians reported seeing > 100 children with asthma per year compared to 63.6% (7/11) of respiratory physicians and 62.5% (5/8) allergists (*p* < 0.001). Just 4% (4/99) of general paediatricians report seeing > 100 children with AR per year compared to 45.5% (5/11) respiratory physicians and 62.5% (5/8) allergists (*p* < 0.001). Furthermore, there was an association between number of children with asthma and/or AR seen and both knowledge of guidelines and experience with prescribing or referring for AIT (*p* = 0.012, *p* = 0.001 and *p* = 0.012 respectively). Respondents who were aware of AR guidelines were more likely to have referred for and prescribed AIT (*p* < 0.001 and *p* = 0.005).

In this study, 30.5% (36/118) of respondents have referred a child for AIT. Allergy or respiratory subspecialisation was associated with referral for AIT with just 24.2% (24/99) of general paediatricians having referred a child, compared to 72.7% (8/11) of respiratory physicians and 50% (4/8) of allergists (*p* = 0.002). The relatively low rate of referral amongst allergists may reflect the fact that 75% (6/8) of them currently work in teams providing AIT and are therefore unlikely to refer patients outside their own service.

AIT has been prescribed by 26.3% (31/118) of respondents. All allergists (8/8) have prescribed AIT along with 63.6% (7/11) of respiratory physicians and 16.2% (16/99) of general paediatricians (*p* < 0.001). Of those who have prescribed AIT, there is an association between specialty and the number of AIT prescriptions written. 87.5% (7/8) of those with an interest/subspecialty in allergy have written > 10 prescriptions and 37.5% (3/8) have prescribed AIT > 50 times. This compares to 45.5% (5/11) of those with a respiratory background and just 12.1% (12/99) of general paediatricians having prescribed AIT > 10 times (*p* = 0.005). 15/118 respondents currently work in a team prescribing AIT. Of these, 11 are consultants and 6/11 are allergists with the remaining 5/11 having a respiratory interest.

### Attitudes towards AIT

77.1% (91/118) of respondents consider SLIT to be effective while 1.7% (2/118) consider it to be ineffective and 21.2% (25/118) were unsure. A large proportion of general paediatricians (25.3%, 25/99) are unsure if SLIT is effective. This compares to 100% of allergists and respiratory physicians.

SLIT is considered safe by 83.1% (98/118) of respondents with 0.8% (1/118) considering it unsafe. The remaining 16.1% (19/118) were unsure. Similarly, 61% (72/118) of respondents consider SCIT to be safe with 2.5% (3/118) considering it unsafe and 36.5% (43/118) were unsure. When those who are unsure are excluded, 99% (98/99) and 96% (72/75) of respondents consider SLIT and SCIT respectively to be safe.

53.4% (63/118) consider SLIT to be cost-effective with 8.5% (10/118) disagreeing. 38.1% (45/118) had no opinion. When these are excluded, 86.3% (63/73) of respondents consider SLIT to be cost-effective. There was an association between current/previous work as part of an AIT team and a negative view of SLIT cost-effectiveness. 17.1% (7/41) of those with AIT team experience consider SLIT not to be cost-effective compared to 3.9% (3/77) of those with no experience (*p* = 0.002).

### Preferred AIT providers

**Fig. 1:**
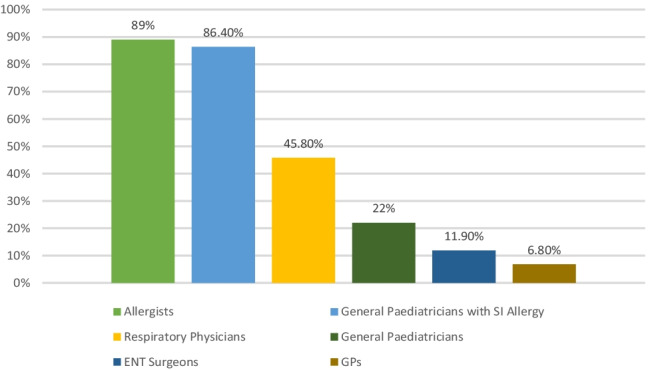
Respondents preference for AIT prescribing and monitoring

The majority of respondents from all specialities and grades believe that AIT should be prescribed and supervised by paediatric allergists or general paediatricians with a special interest in allergy (see Fig. 1). This was followed by respiratory specialists, ENT surgeons, general paediatricians and finally GPs. Only 30% (3/10) of respiratory consultants felt that AIT should be prescribed by their team despite 50% (5/10) of them providing AIT at the time of the study. 90% (9/10) of respiratory consultants believe that AIT should be prescribed by allergists and 80% (8/10) by general paediatricians with an interest in allergy.

### Barriers to AIT in Ireland

**Fig. 2:**
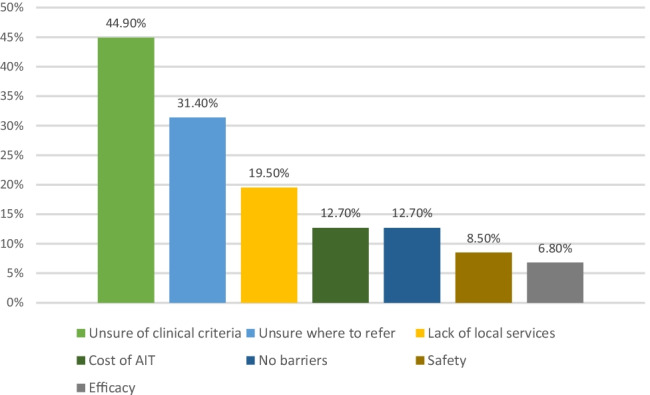
Barriers to prescribing and referral for AIT in Ireland

Figure 2 illustrates the major barriers to AIT in Ireland identified by this study.

A lack of knowledge of AIT clinical criteria was frequently reported with 50.5% (50/99) of general paediatricians unaware of the indications for referral compared to 27.3% (3/11) of respiratory physicians and 0% (0/8) of allergists (*p* = 0.006). This lack of knowledge was recognised by respondents as 115/118 (97.5%), including all general paediatricians, stated that they would like to know more about AIT.

Accessibility is the next most cited barrier with 31.4% (37/118) of respondents unsure where to refer and 19.5% (23/118) reporting a lack of local services. This barrier affects general paediatricians only, with 37.4% (37/99) reporting this while all allergists and respiratory physicians are aware of referral pathways (*p* = 0.003).

## Discussion

Despite the high prevalence of AR amongst Irish children, the use of AIT remains low [13, 14, 18]. The response rate of 41.4% exceeded our target and the results are felt to be representative of the views of Irish paediatricians.

The majority of respondents reported favourable views of AIT in terms of efficacy, safety and cost. There is a preference for AIT to be prescribed and supervised by allergists/general paediatricians with a special interest in allergy. These findings are similar to a study of Turkish paediatricians upon which our questionnaire was based [23].

A Europe-wide study looking at barriers to implementation of EAACI AIT guidelines identified accessibility and cost as being the main barriers in Ireland [22]. This study, which was based on a larger sample size, identified lack of knowledge amongst general paediatricians as the main barrier to AIT. The second largest barrier is accessibility of AIT, with a lack of local services and referral pathways. In contrast, cost was listed as a barrier by just 12.7% (15/118) of respondents, though was associated with previous or current experience of prescribing AIT. This may reflect the practical experience of patients without a medical card having to pay over €1000 per year for AIT.

General paediatricians in particular identified accessibility as a barrier with 37.4% (37/99) reporting this. This is despite a study from 2012 which reported AIT being available in 14 centres across the country [18]. This discrepancy may reflect a reduction in the availability of AIT services during the intervening years or the small overall numbers of physicians providing AIT services with just 11 consultants in this study currently providing AIT.

This study demonstrated differences between the opinions of Irish respiratory specialists and those in Europe. While they demonstrated a good level of knowledge and favourable attitudes towards AIT, just 45.5% of respiratory specialists that responded currently prescribe AIT and an additional 27.3% have previous experience doing so. This is significantly less than the 78% of Italian chest physicians prescribing AIT reported by Lombardi [24]. Just 30% of Irish respiratory specialists believe they should be prescribing AIT with 90% preferring AIT to be supervised by paediatric allergists.

This study has identified a group of paediatricians with a special interest or sub-specialisation in paediatric allergy or respiratory who see large numbers of children with asthma and allergic rhinitis. These doctors typically have experience of current or previous work with an AIT team and are aware of international guidelines. They are aware of the local services available and prescribe or refer many children for AIT. In contrast, many general paediatricians are unaware of what patients are suitable for AIT and where to access this treatment.

This study demonstrated positive attitudes towards AIT amongst all grades and specialities of paediatricians in Ireland despite low rates of AIT prescribing. The main barriers to use are difficulties with the identification of suitable candidates by general paediatricians and an actual or perceived lack of local AIT services and referral pathways. This study highlights the need for educational sessions for general paediatricians and paediatric trainees to ensure that this treatment is considered for suitable patients. Further work should focus on quantifying the current availability of AIT services nationally to investigate the change in availability since the previous survey in 2012 and to ensure local referral pathways are put in place. This study is the first to evaluate the knowledge and attitudes of paediatricians in Ireland towards AIT and will be useful in the planning and delivery of AIT services nationally.
